# A lyophilized colorimetric RT-LAMP test kit for rapid, low-cost, at-home molecular testing of SARS-CoV-2 and other pathogens

**DOI:** 10.1038/s41598-022-11144-5

**Published:** 2022-04-29

**Authors:** Xin Song, Felicity J. Coulter, Ming Yang, Jessica L. Smith, Fikadu G. Tafesse, William B. Messer, John H. Reif

**Affiliations:** 1 Department of Electrical and Computer Engineering, Duke University, Durham, NC 27708 USA; 2 Department of Biomedical Engineering, Duke University, Durham, NC 27708 USA; 3 Department of Computer Science, Duke University, Durham, NC 27708 USA; 4 Department of Molecular Microbiology and Immunology, Oregon Health and Science University, Portland, OR 97239 USA; 5 Vaccine and Gene Therapy Institute, Oregon Health and Science University, Beaverton, OR 97006 USA; 6 Department of Medicine, Division of Infectious Diseases, Oregon Health and Science University, Portland, OR 97239 USA; 7 Program in Epidemiology, OHSU-PSU School of Public Health, Oregon Health and Science University, Portland, OR 97239 USA

**Keywords:** Biochemistry, Biological techniques, Biotechnology, Infectious diseases, Infectious-disease diagnostics

## Abstract

Access to fast and reliable nucleic acid testing continues to play a key role in controlling the COVID-19 pandemic, especially in the context of increased vaccine break-through risks due to new variants. We report a rapid, low-cost (~ 2 USD), simple-to-use nucleic acid test kit for self-administered at-home testing without lab instrumentation. The entire sample-to-answer workflow takes < 60 min, including noninvasive sample collection, one-step RNA preparation, reverse-transcription loop-mediated isothermal amplification (RT-LAMP) in a thermos, and direct visual inspection of a colorimetric test result. To facilitate long-term storage without cold-chain, a fast one-pot lyophilization protocol was developed to preserve all required biochemical reagents of the colorimetric RT-LAMP test in a single microtube. Notably, the lyophilized RT-LAMP assay demonstrated reduced false positives as well as enhanced tolerance to a wider range of incubation temperatures compared to solution-based RT-LAMP reactions. We validated our RT-LAMP assay using simulated infected samples, and detected a panel of SARS-CoV-2 variants with successful detection of all variants that were available to us at the time. With a simple change of the primer set, our lyophilized RT-LAMP home test can be easily adapted as a low-cost surveillance platform for other pathogens and infectious diseases of global public health importance.

## Introduction

The COVID-19 pandemic has cost millions of lives and presented unprecedented economic, social, and structural challenges across the world. Whilst vaccination rollouts and improved access to testing have helped to control the pandemic^1^, transmissions and infections (including post-vaccine breakthrough cases) due to the new variants of SARS-CoV-2^2,3^ continue to pose a burden on global public health and economics. Effective SARS-CoV-2 surveillance requires frequent testing with rapid results to quickly identify infected individuals to break transmission chains^4^. However, centralized testing models such as drive-through tests may increase the risk of exposure to health care workers and also rely on costly facilities, trained personnel, and sophisticated lab equipment (typically RT-qPCR) that in many cases still fail to deliver a timely test result. These delays can be detrimental to effective surveillance and disease control efforts due to the risk of presymptomatic/asymptomatic transmission of SARS-CoV-2^5^. While testing labs are widely available in many high-income countries, most low- and middle-income countries lack sufficient facilities and trained personnel for wide-spread application of sophisticated SARS-CoV-2 detection technologies. Rapid antigen tests are easy to use and less expensive, however, while they are effective at screening symptomatic patients with high viral loads^6^, the overall higher rates of false positives and false negatives (compared to nucleic acid tests) make rapid antigen tests less suitable as a front-line diagnostic^7,8^. To quickly identify emerging SARS-CoV-2 transmission hotspots and curb the spread of virus from all potential transmission routes (presymptomatic, symptomatic, asymptomatic), a robust decentralized testing model would require the development of affordable nucleic acid home tests that are reliable, simple to use, and inexpensive to manufacture and distribute to large populations.

Since the beginning of the pandemic, researchers have sought to develop rapid molecular assays to overcome the practical limitations of standard RT-qPCR testing^9^. Among several candidate nucleic acids amplification protocols^10^, RT-LAMP is a simple method that achieves rapid exponential amplification of RNA using a set of six primers to recognize eight distinct regions on the target RNA sequence, enabling highly specific and sensitive detection of target RNA without stringent requirement on sample purity^11^. This eliminates the need for the sophisticated RNA isolation and purification processes that have been a major bottleneck of current SARS-CoV-2 testing workflows. Further, the compatibility with simple pH-based colorimetric readout^12^ allows easy interpretation of the test result by visual inspection, making RT-LAMP suitable for inexpensive point-of-care applications. To date, many RT-LAMP assays have been proposed for SARS-CoV-2 detection^13–22^ including several that have obtained FDA Emergency Use Authorization (EUA)^23–25^. However, most of these tests still cannot meet the need for frequent at-home testing due to either un-optimized performance or the prohibitive cost per test.

## Results

### Assay development

**Figure 1:**
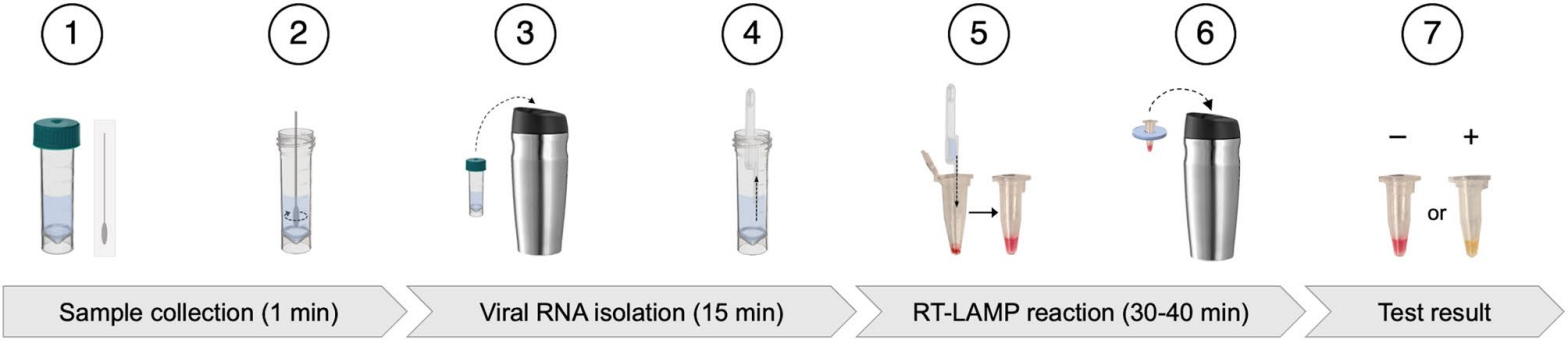
Schematic illustration of the COVID-19 home test from sample collection to test result readout. Step 1: Self-collect a sample using an anterior nasal swab (alternatively a gingival swab). Step 2: Plunge the swab into the media inside the collection tube. Gently rub and roll the swab against the tube wall for 10 times. Squeeze out the remaining liquid by pressing the swab against the side of the tube, discard the swab and recap the tube. Step 3: Add 95 °C hot water into a thermos. Ensuring the sample collection tube lid is tightly secured, place inside the thermos and close the lid, to incubate for 10 min. Step 4: Take out the collection tube and cool it on ice for 5 min, allowing any debris to settle to the bottom. Use a disposable transfer pipette to draw 20 μL of sample from the collection tube. Step 5: Quickly dispense the sample into the lyophilized RT-LAMP reaction tube. Recap and gently flick the side of the reaction tube to resuspend the mix (avoid introducing bubbles). Step 6: Use a thermometer or a temperature sticker (included in the kit) to adjust the water temperature to 65 °C in the thermos. Assemble the RT-LAMP reaction tube onto the foam floater and place it in the thermos. Close the lid and incubate for 40 min. Step 7: Take out the reaction tube and cool it on ice. Visually inspect the test result (pink = negative; yellow = positive).

Conventional molecular assays do not support convenient use at home by untrained individuals because of the complexity of the testing workflow, dependence on specialized instrumentation, stringent requirement of cold storage for reagents, and the high cost of the test platform manufacturing and distribution. To enable truly inexpensive, rapid, reliable at-home testing of COVID-19, we developed a simple all-in-one molecular home test kit based on lyophilized colorimetric RT-LAMP, requiring only a regular thermos and a thermometer to conduct the self-administered test. A rapid, one-pot lyophilization protocol was developed to quickly preserve all reagents needed for the colorimetric RT-LAMP test in a single microtube, facilitating long-term stability, inexpensive distribution, and convenient use of the home test kit. Notably, the lyophilized RT-LAMP assay demonstrated reduced false positives and higher tolerance to a wider range of incubation temperatures compared to conventional solution-based RT-LAMP reactions. To enable detection of viruses from clinical sample matrices, we adapted a one-step RNA preparation protocol^19^ based on low-cost shelf-stable reagents. The entire sample-to-answer workflow (Fig. 1) takes < 60 min, including noninvasive sample collection (anterior nasal swab or alternatively gingival swab), quick extraction-free RNA preparation, optimized RT-LAMP reaction in a thermos, and finally a colorimetric interpretation of the test result.

RT-LAMP reactions rely on active enzymatic components (i.e., DNA polymerase and reverse transcriptase) that must be stored at a low temperature (typically − 20 °C). To preserve the RT-LAMP reagents for home test use, we employed lyophilization, also known as freeze-drying, to extend the shelf-life of the test kit and facilitate simple test kit distribution, handling, and storage under convenient temperatures (e.g., at typical home-refrigeration temperature or at room temperature). A lyophilized test kit also reduces the number of pipetting steps to improve usability and minimize contamination^26^. However, lyophilization is typically an expensive and time-consuming process involving three stages including freezing, primary drying, and secondary drying which can be difficult to design and optimize^27^. In this work, we developed a fast, one-pot lyophilization process that minimizes the drying time by completing both the primary and secondary drying under a single condition^28^. Unlike prior lyophilization protocols developed for molecular biology assays, our protocol eliminates the need to separately lyophilize the reaction buffer and the enzymes^17,26,29^. Instead, our simplified protocol enables one-pot lyophilization of all reagents needed for the colorimetric RT-LAMP in a single microtube (Table 1), and the entire lyophilization process can be completed in under 2 h (see Methods). We tested trehalose^30–32^ and dextran^33,34^ as candidate excipients to provide cryo- and lyoprotection during lyophilization, as well as enhanced stability for long-term storage. In addition, guanidine hydrochloride (GuHCl)^35^ was included in the optimized formulation to improve the reaction speed and the sensitivity of colorimetric RT-LAMP. We carefully screened multiple sets of recently published RT-LAMP primers^14,15,36–38^ and optimized our RT-LAMP assay with a well-performing primer set^38^ (Table 2), which targets the ORF1a gene of the viral genome and is minimally impacted by the mutations from recent SARS-CoV-2 variants.

**Table 1:** Optimized formulation of the lyophilized colorimetric RT-LAMP test.

Component	Amount used per kit
WarmStart^®^ colorimetric LAMP 2X master mix	10 µL
10X primer mix	2 µL
3 M Trehalose	3 µL
1 M GuHCl	0.8 µL

**Table 2:** SARS-CoV-2 RT-LAMP primer set used in the optimized RT-LAMP test.

Primer	Sequence 5′ → 3′
F3	CGGTGGACAAATTGTCAC
B3	CTTCTCTGGATTTAACACACTT
FIP	TCAGCACACAAAGCCAAAAATTTATTTTTCTGTGCAAAGGAAATTAAGGAG
BIP	TATTGGTGGAGCTAAACTTAAAGCCTTTTCTGTACAATCCCTTTGAGTG
LoopF	TTACAAGCTTAAAGAATGTCTGAACACT
LoopB	TTGAATTTAGGTGAAACATTTGTCACG

Compared to typical solution-based RT-LAMP reactions, we observed that the addition of trehalose at the optimized concentration significantly reduced the occurrence of RT-LAMP false positives. The slight decrease in reaction speed was mitigated by the addition of GuHCl. Notably, our lyophilized RT-LAMP reactions also enabled a wider compatible range of incubation temperatures compared to the solution-based RT-LAMP reactions, thus improving the assay’s robustness to tolerate the use of regular thermoses for reaction incubation without precise temperature control. Specifically, as shown in Fig. S7, our lyophilized assay (“3 M trehalose + 1 M GuHCl) lyo”) performed robustly across the entire temperature gradient tested (60.7–70.0 °C) with clear readout of true positives as early as 20 min and no false positives by 50 min of incubation at most temperatures within the temperature gradient. In contrast, the solution-based RT-LAMP assay (“Fresh sol”) based on the same primers and master mix formulation showed a narrower range of compatible temperatures, slower turnaround, and earlier occurrence of false positives. The beneficial effect of the one-pot lyophilization was also observed in Fig. S15, where we conducted a similar temperature gradient experiment for the assay based on a different published RT-LAMP primer set^23^. We hypothesize that the enhanced performance of the one-pot lyophilized assay is partly due to the inclusion of trehalose in the RT-LAMP formulation. In addition to its role as a lyo- and thermal-protectant, trehalose was found to have a DNA duplex destabilizing effect in prior literature^39^. Such an effect helps to improve the specificity and yield of isothermal amplification reactions, which agrees with the observations from our experiments.

### Assay validation

**Figure 2:**
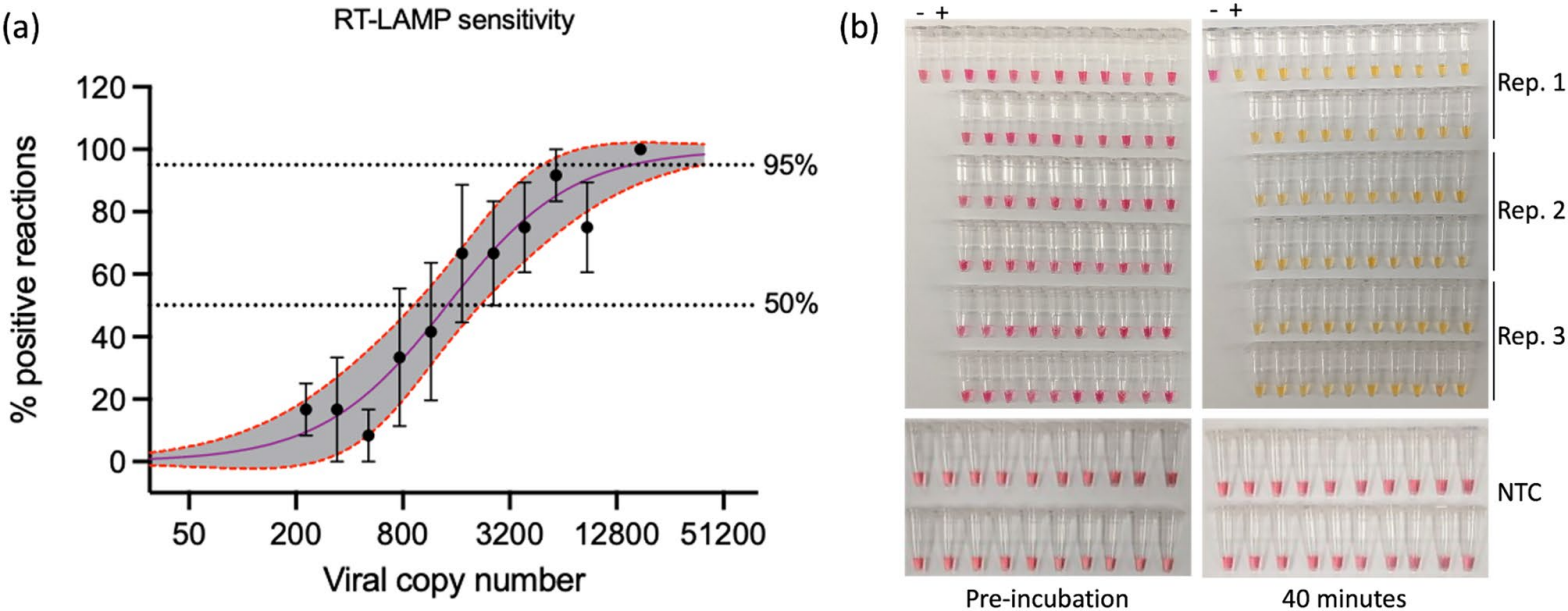
Limit of detection and validation of analytical sensitivity and specificity on virus-spiked anterior nasal swab collection media. (**a**) Serial dilutions of heat-inactivated SARS-CoV-2 strain WA-1/2020 by percent positive samples. Three independent replicates of four-reactions per dilution were performed using samples from 227 copies per reaction to 17,473 copies per reaction. X-axis is log-scale showing copies per reaction, Y-axis shows % positive reactions. Each data point shows the average and standard error of the mean (SEM) for each set of replicates per dilution. A Sigmoidal dose–response curve was fit using the Find ECAnything least squares fit in Graphpad Prism 7.0 with lower and upper limits of 0 and 100 respectively. Red-dotted lines and grey area indicates 95% confidence interval around the dose–response curve. Straight dotted lines show 50 and 95% analytic sensitivity intercepts as predicted by the fitted curve. 95% limit of detection was 15,514 viral copies, 50% limit of detection was 1421 viral copies per reaction. (**b**) 20 replicates of non-template control (NTC, anterior nasal swab media only; bottom panels) versus 15,518 viral copies of heat-inactivated SARS-CoV-2 (top panels) in uninfected pooled anterior nasal swab media, demonstrating ≥ 99% analytical specificity and ≥ 95% analytical sensitivity at 15,518 copies/reaction. Nuclease-free water non-template control (−) and BEI RNA positive control (+), indicated. Pre-incubation and incubation times and colorimetric changes as above.

According to in-house validation using synthesized SARS-CoV-2 RNA (Twist Bioscience), our test kit remains stable for at least 30 days at typical home-refrigeration temperature (4 °C) (Fig. S11) and 10 days at room temperature (~ 20 to 22 °C) (Fig. S14), achieving ≥ 95% analytical sensitivity and > 99% specificity with a reproducible limit of detection (LoD) down to 100 RNA copies per reaction (i.e., 5 copies/μL) under both storage conditions. In addition, we conducted validation tests using simulated SARS-CoV-2 infected samples—heat-inactivated SARS-CoV-2 WA-1 virus serially diluted and spiked into pooled anterior nasal swab media (0.0025X TBE buffer in nuclease-free water)—from healthy donors (Fig. 2a). Sensitivity for authentic SARS-CoV-2 WA-1/2020 was significantly reduced in comparison to the sensitivity under laboratory conditions with commercially purified RNA, 15,514 viral copies per reaction, but at higher viral loads the assay demonstrated reliable sensitivity, specificity, and reproducibility for detection of SARS-CoV-2 virus (Fig. 2b). Specificity remained at ≥ 99% for the simulated samples (Fig. 2b). Aliquots of simulated SARS-CoV-2 infected samples were also subjected to qRT-PCR in the OHSU clinical lab using the Fisher Multiplex TaqPath platform with 15,514 viral copies returning a calculated CT value of 26.4. We next examined the distribution of CT values for 5897 samples tested across symptomatic and asymptomatic patients tested in the OHSU clinical lab (data provided generously by Dr. Xuan Qin, OHSU Department of Pathology), finding that the RT-LAMP assay would be expected to have detected SARS-CoV-2 in approximately 4254 patients, the number of patients with a CT of < 26, yielding a real-world sensitivity of 72%.

**Figure 3:**
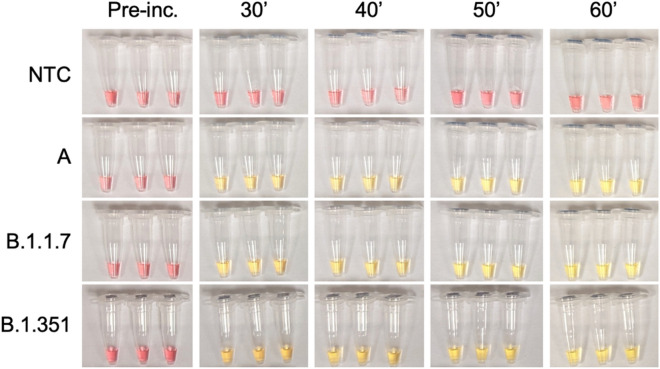
Validation of the RT-LAMP home test on heat inactivated SARS-CoV-2 variants (calculated viral copy numbers per tube indicated): A (WA-1/2020; 4.86 × 10^4), B.1.1.7 (1.51 × 10^5), and B.1.351 (4.79 × 10^5). Heat-inactivated virus was diluted in nuclease-free water. Viral copy numbers were calculated following quantification of stocks. Methods otherwise as described previously. NTC: non-template control (nuclease-free water).

**Figure 4:**
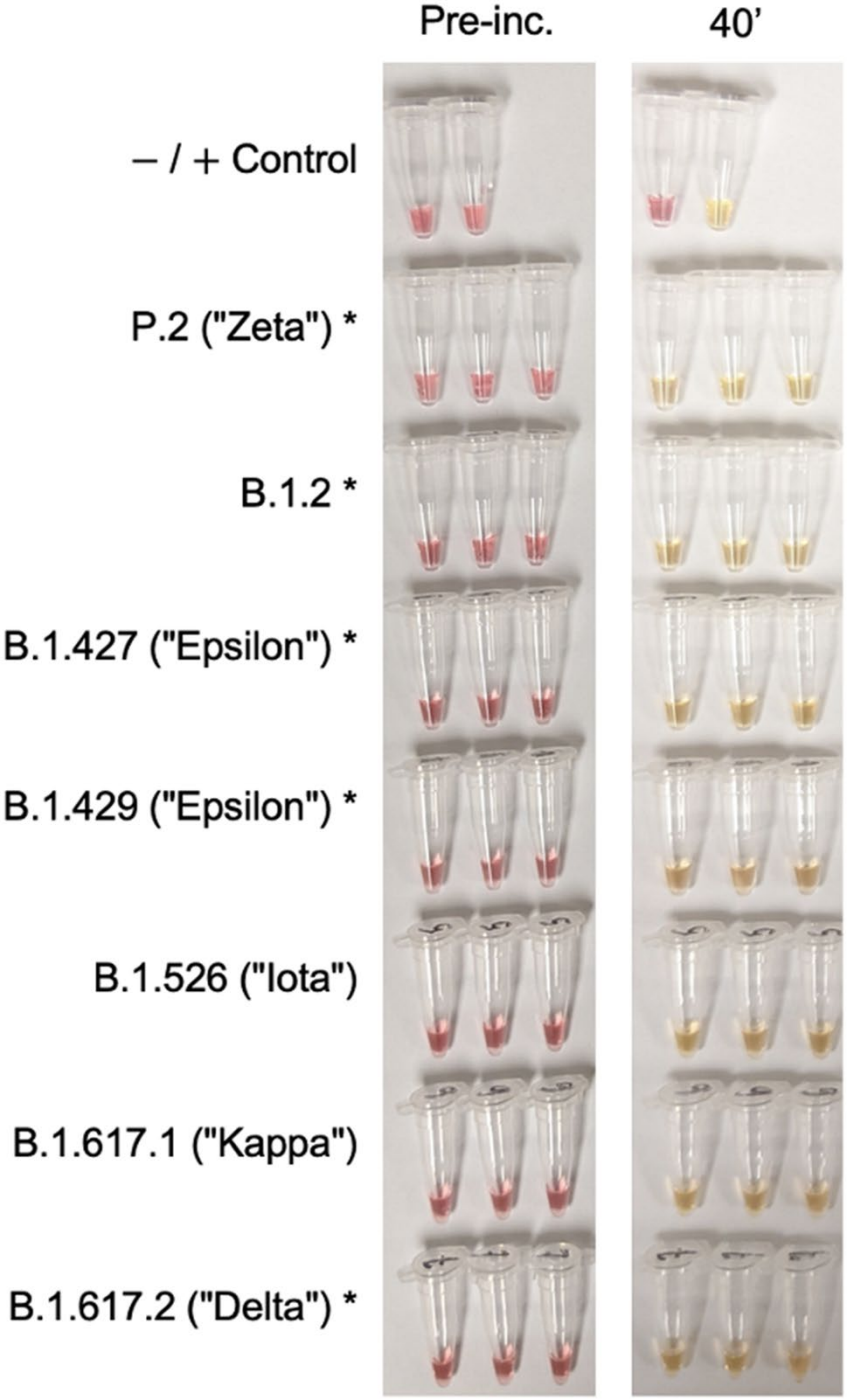
Validation of the RT-LAMP home test on additional heat inactivated SARS-CoV-2 variants (calculated viral copy numbers per tube): P.2 (6.60 × 10^4), B.1.2 (2.69 × 10^5), B.1.427 (5.05 × 10^5), B.1.429 (4.12 × 10^4), B.1.526 (2.72 × 10^5), B.1.617.1 (1.65 × 10^5) and B.1.617.2 (4.05 × 10^4). Negative and positive controls were nuclease-free water and commercially available genomic RNA (BEI), respectively. Asterisk indicates viruses originating from clinical samples, confirmed by sequencing.

Like most RNA viruses, SARS-CoV-2 has and will continue to evolve genetically, and there are now several genetic variants of SARS-CoV-2, which the U.S. Centers for Disease Control and Prevention classifies as Variants Being Monitored (VBM), Variants of Interest (VOI), Variants of Concern (VOC) or Variants of High Consequence (VOHC). Classification depends on the presence of substitutions that may confer increased transmissibility, disease severity, immune and therapeutic escape, and interference with diagnostic test targets. It is thus important to assess the accuracy of our assay in detecting newly emerged SARS-CoV-2 variants. After carefully screening multiple sets of recently published RT-LAMP primers^14,15,36–38^, we optimized our RT-LAMP test with a well-performing primer set (Table 2)^38^ targeting the ORF1a gene of the SARS-CoV-2 viral genome. As shown in Figs. 3 and 4, our test successfully detected multiple SARS-CoV-2 variants and their isolates from different geographical locations, including WA1/2020 (USA), B.1.1.7 (“Alpha", UK), B.1.1.7 (“Alpha”, US-CA), B.1.351 (“Beta”, South Africa), P.2 (“Zeta”, Brazil), B.1.2 (US-LA/NM), B.1.427 (“Epsilon”, US-CA), B.1.429 (“Epsilon”, US-CA), B.1.526 (“Iota”, US-NY), B.1.617.1 (“Kappa”, India), and B.1.617.2 (“Delta”, India). The simplicity of our assay allows quick change of the primer sets to detect emerging variants of SARS-CoV-2 and other pathogens and diseases of public health importance.

## Discussion

**Figure 5:**
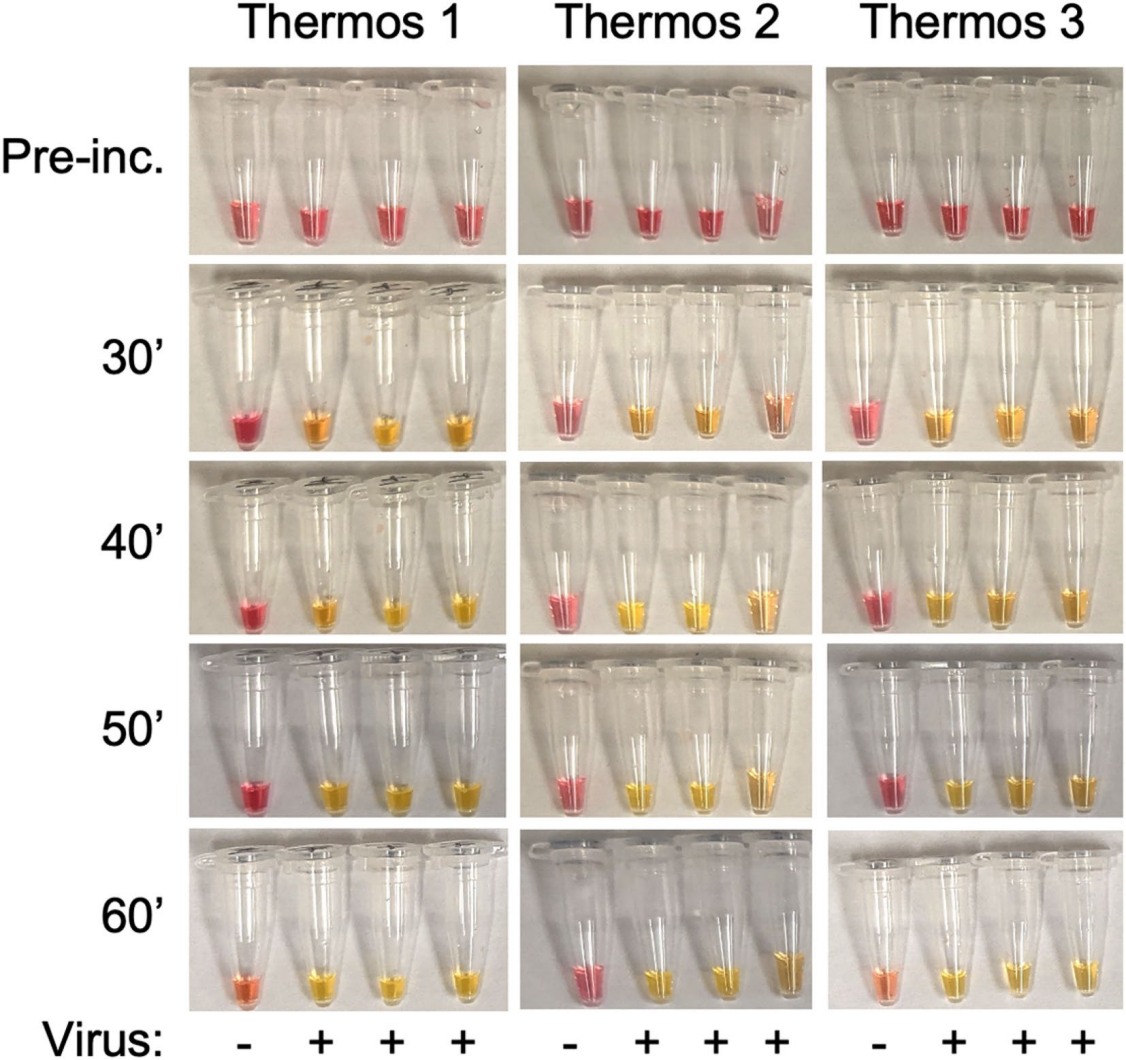
Validation of the RT-LAMP home test in different commercially available thermoses (see Methods for thermos details. Viral strain: Inactivated SARS-CoV-2 A (WA-1/2020) in gingival swab media. One non-template control (gingival swab media only) and triplicate virus, (−) and ( +) respectively, per thermos experiment.

In this work, we have demonstrated an inexpensive, one-pot lyophilized colorimetric RT-LAMP molecular test kit for self-administered COVID-19 diagnosis. In addition to its low cost and simplicity, the test kit features a user-friendly home testing workflow that can be easily completed in under 1 h with no specialized instrumentation or trained personnel (Table 3). Specifically, we developed a simple one-pot protocol for lyophilizing colorimetric RT-LAMP. All reagents needed for the isothermal amplification reaction can be quickly preserved in a single microtube, facilitating long-term storage, inexpensive distribution, and simple testing workflow without multiple liquid transfers. Unlike prior work of lyophilized LAMP/RT-LAMP that requires sophisticated lab procedures to separately lyophilize the enzymes from the reaction buffers, the simplicity and robustness of our one-pot lyophilization protocol makes it easy to inexpensively manufacture the molecular test kits at scale. We tested our RT-LAMP assay in regular thermoses and verified its tolerance to temperature deviations in different thermoses (Fig. 5). Notably, our test conveniently tolerates larger sample input volumes (i.e., as opposed to 1 μL to 5 μL sample volume commonly used in standard RT-LAMP assays, our test directly accepts 20 μL swab media sample to rehydrate the lyophilized reagents for a 20 μL RT-LAMP reaction). Furthermore, in contrast to conventional molecular diagnostics that usually involve multiple precise volume liquid transfers, our test requires only a single pipetting step (using a low-cost disposable transfer pipette) during the entire testing workflow.

**Table 3:** Features and advantages of the COVID-19 molecular home test kit.

Low cost	Total cost per all-in-one kit ~ 2 USD (detailed cost breakdown in Table S1)
Simple, noninvasive, reliable workflow	No need for standard/specialized lab equipment or trained personnel Self-sample collection with minimal discomfort (anterior nasal swab or gingival swab) Simple and reproducible testing workflow with minimal liquid transfer steps Tolerant of temperature deviations in regular thermoses
Rapid and visual result	Sample-to-result in under 1 h Simple visual inspection of colorimetric test readout
Sensitive and accurate RT-LAMP assay	Tolerant to large sample input volume: 20 μL sample directly reconstitutes RT-LAMP Excellent analytical limit of detection: 100 RNA copies per reaction (i.e., 5 copies/μL) Excellent analytical sensitivity: ≥ 95% Excellent analytical specificity: > 99% Detects all SARS-CoV-2 variants that were tested in this paper
Long-term stability	Stable for ≥ 30 days at typical home-refrigeration temperature (4 °C) Stable for ≥ 10 days at room temperature (~ 20–22 °C)
Easy to manufacture	Fast (2 h) one-pot lyophilization preserves all test regents in a single microtube Extraction-free RNA preparation with inexpensive shelf-stable reagents
Easy to distribute	Compact all-in-one home test kit Minimal accessories required (thermos, thermometer, ice)* Good stability for potential over-the-counter distribution without cold chain

*The thermos/thermometer can be replaced by other low-cost solutions depending on use case.

Nevertheless, the present work has several limitations. First, the RT-LAMP assay is qualitative and relies on visual interpretation of the colorimetric test result. Besides potential user errors, extended storage of the current test kit under elevated temperatures may reduce the color contrast between the positive and negative readouts, thus increasing the likelihood of misinterpretation. The continuing development of the test kit requires thorough characterization and rigorous testing of the assay performance under uncontrolled environments, for example, by conducting shipping and storage simulations under a wider range of temperature and humidity conditions. Second, while we have demonstrated excellent analytical performance of the underlying lyophilized RT-LAMP assay, further optimizations of the RNA isolation protocol are needed to achieve better sensitivity on clinical samples. However, for perspective, we compared the results of our dose–response evaluation of the RT-LAMP platform in its current configuration with the real-world CT values from the OHSU clinical diagnostic laboratory, we predict a real-world sensitivity of approximately 72% for all SARS-CoV-2 infections. While this value is below the performance of some other RT-LAMP platforms, it is well above the predicted sensitivity for symptomatic individuals of the widely used BinaxNOW for symptomatic individuals of 64.2%^40^. Third, the off-the-shelf RT-LAMP master mix used in the present assay contains glycerol, which may cause insufficient removal of water during lyophilization^41^. While our real-time stability experiments suggested that the simple one-pot lyophilization could readily preserve the colorimetric RT-LAMP reactivity for > 4 months (data not shown), the long-term stability of our assay could be substantially improved by using glycerol-free enzymes. Fourth, the present study evaluated an extraction-free RNA preparation protocol (i.e., heating for 10 min at 95 °C in 0.0025X TBE) but did not assess its efficacy for SARS-CoV-2 inactivation. While recent studies^19,42^ have suggested the use of similar heating protocols for effective inactivation of SARS-CoV-2, thorough validations must be conducted before pursuing other applications beyond home testing.

Compared to other COVID-19 testing platforms available at the time of this writing, our rapid molecular test kit shows good potential to enable affordable and frequent at-home testing. Due to its low cost and simplicity, our test can allow mass manufacturing in a short timeframe to potentially address the pressing need for global population-scale surveillance, especially in resource-limited regions where COVID-19 is still raging and vaccinations are lagging. For users who cannot conveniently perform the test at home, our test kits can also be readily used at point-of-care settings such as local pharmacies or mobile laboratories, where batch testing of samples can be easily conducted on site using a dedicated dry or water bath or a similar heat source. The patient would still self-collect a sample using the provided swab with the collection tube and then return the sample to the pharmacy. Due to the fast turnaround of our RT-LAMP assay, the test result can be returned to the patient in under one hour. Advantages of using our tests for point-of-care/near-patient testing include the further simplified testing workflow and the improved quality control of the tests. However, this alternative configuration would require a technician to handle the patient samples, thus enhanced precautions must be carefully followed (e.g., use of PPE, hand hygiene, frequent instrument decontamination) and assure that all samples are fully inactivated upon receipt. Ideally, the sample handling protocol would be automated at the pharmacy to reduce the risk and improve the testing throughput. Furthermore, it is important to note that our all-in-one lyophilized colorimetric RT-LAMP test kit can be quickly adapted to detect different RNA or DNA targets by simply changing the primer set. This remarkable flexibility coupled with the simplicity and reliability of our test kit and testing workflow hold great promise to enable a robust model platform for low-cost decentralized surveillance of other pathogens (e.g., viruses, bacteria, fungi), including infectious diseases of global public health importance (e.g., dengue, tuberculosis, malaria).

## Methods

### RT-LAMP primers

Several published SARS-CoV-2 RT-LAMP primer sets^14,15,36–38^ were carefully screened in terms of the detection sensitivity, false positive and false negative rates, reaction speed, and test reproducibility (Figs. S1–S6). The best performing primer set^38^ (Table 2) was selected for further characterization and optimization in our lyophilized colorimetric RT-LAMP home test kit. This primer set targets the ORF1a gene of the SARS-CoV-2 viral genome and is minimally impacted by mutations on recent SARS-CoV-2 variants of concern. An additional primer set^23^ (Table S2) was tested to confirm the reliable performance of the one-pot lyophilization protocol.

### RT-LAMP reagents

WarmStart Colorimetric LAMP 2X Master Mix (New England Biolabs, cat. M1800L) was used as the RT-LAMP master mix for the test kit. RT-LAMP primers were ordered from IDT as custom DNA oligos with standard desalting. The primers were resuspended in nuclease-free water (Sigma-Aldrich) and mixed to form a 10X primer mix consisting of 2 µM F3 primer, 2 µM B3 primer, 16 µM FIP primer, 16 µM BIP primer, 4 µM LoopF primer, and 4 µM LoopB primer. RT-LAMP reactions were run at 20 µL total reaction volume. Specifically, the lyophilized RT-LAMP reagents were reconstituted with 5 µL sample + 15 µL nuclease-free water in all analytical experiments conducted with synthetic SARS-CoV-2 RNA control (Twist Bioscience, cat. 102024). Unless otherwise specified, 20 µL of sample (as opposed to 5 µL sample + 15 µL nuclease-free water) was directly added to the lyophilized RT-LAMP mix in validation experiments conducted with simulated SARS-CoV-2 infected samples.

### Fast one-pot lyophilization of colorimetric RT-LAMP

The 3 M trehalose solution was prepared by dissolving 0.5 g D-(+)-trehalose dihydrate powder (Sigma-Aldrich, M.W. 378.33 g/mol) in 440.5 μL nuclease-free water, followed by vigorous vortexing and heating at 60 °C for 10 min to fully dissolve the trehalose to yield a supersaturated solution. This resulted in a solution with a total volume of approximately 760 μL, corresponding to an effective trehalose concentration of around 1.75 M. Because our assay prototyping needed only small amounts of the solutions, to keep the measuring simple and consistent, we refer to this resulting solution as the 3 M trehalose throughout the text unless otherwise specified. The trehalose solution was then sterilized by filtering through a 0.2 µm syringe filter (VWR), followed by brief vortex and centrifuge to remove air bubbles. For consistency, the 1 M GuHCl solution was prepared similarly by directly dissolving 0.1 g guanidine hydrochloride powder (VWR, M.W. 95.53 g/mol) in 1046.8 μL nuclease-free water without further adjustment of the final volume. The tube containing the GuHCl solution was covered with aluminum foil to protect it from light. Components of the colorimetric RT-LAMP lyophilization formulation were mixed at the specified ratio (Table 1), aliquoted into 0.2 mL PCR tubes, and frozen at − 20 °C for 1 h. Finally, the tubes were quickly transferred with caps open into a vacuum concentrator (Savant Speedvac SVC-100H) connected to the lyophilizer (VirTis Freezemobile 12SL). Lyophilization was run for 1 h with the chamber pressure at ~ 10 milliTorr and the condenser temperature at ~  − 40 °C. Details of the RT-LAMP lyophilization protocol optimizations are shown in Fig. S7–S16.

### Viruses

SARS-CoV-2 Pango lineage A (WA-1/2020) was used in all whole virus assays for in-house test kit optimization. Other variants obtained through BEI Resources were B.1.1.7, B.1.351, B.1.526.2 and B.1.617.1. Other viruses were isolated from clinical samples, and lineage determined by next generation sequencing. These include P.2, B.1.2, B.1.427, B.1.429, B.1.526, and B.1.617.2. Further virus information including source are summarized in Table S3. Authentic SARS-CoV-2 were propagated and inactivated in a BSL-3 laboratory under a protocol approved by the OHSU Institutional Biosafety Committee under the supervision of Dr. Tafesse.

### Simulated SARS-CoV-2 infected samples

Anterior nasal swab (and alternatively gingival swab) samples were collected as described below from 10 uninfected individuals, into sample collection media (TBE (1X: 90 mM Tris–borate-2 mM EDTA, pH 8.0) at the specified concentration, in nuclease-free water) and pooled. Heat-inactivated SARS-CoV-2 virus was diluted in sample collection media to achieve the specified viral copy numbers, according to in-house qRT-PCR. Virus-spiked samples were heated at 95 °C for the specified duration and chilled on ice. Then, 20 µL of the resulting sample was added directly into the RT-LAMP microtube to reconstitute the lyophilized reagents. RT-LAMP microtubes were vortexed (spun down, if necessary) and briefly chilled on ice, before pre-incubation photos were taken. Finally, RT-LAMP microtubes were incubated for 60 min at 65 °C, with photos taken at 30–60 min to assess color change. The best detection sensitivity was achieved by RNA isolation with 0.01X TBE (based on 5 µL sample input into a 20 µL reaction) and heating for 10 min at 95 °C (Figs. S17–18). Subsequent assays were performed using 20 µL direct sample input and 0.0025X TBE, to reduce pipetting steps.

### Colorimetric RT-LAMP in thermocycler

RT-LAMP microtubes containing samples or non-template control (NTC) were vortexed, spun down and briefly chilled on ice before pre-incubation photos were taken. RT-LAMP microtubes were incubated in a thermocycler for 60 min at the specified temperature, with photos taken at 30–60 min to assess color change. Tubes were briefly chilled on ice to allow color stabilization, before being photographed. The optimal RT-LAMP incubation temperature was identified by running the reactions with a temperature gradient (T = 65 °C, G = 5 °C) set in a gradient thermal cycler (Eppendorf MasterCycler). To avoid contamination, the RT-LAMP tubes should never be reopened after the incubation reaction.

### Colorimetric RT-LAMP in thermos

Both the viral RNA preparation and the RT-LAMP incubation were conducted in a thermos. Freshly boiled water was added to pre-warm the thermos for 2 min and then dumped out. Next, boiling water was re-added into the thermos and chilled to ~ 97 °C, after which the virus-spiked samples and NTC (swab media without virus) were incubated for 10 min in the thermos (with lid on) and then chilled on ice for 5 min to allow cell debris to settle. Next, 20 µL of the heat-inactivated sample “supernatant” (i.e., no cell debris) or NTC were transferred to the RT-LAMP microtube using a disposable transfer pipette. The microtubes were recapped and flicked gently (being careful not to introduce bubbles) to resuspend the lyophilized RT-LAMP reagents and then chilled on ice before pre-incubation photos were taken. Meanwhile, a mug was filled with boiling water, and allowed to chill to ~ 70 °C before pouring into the thermos. The water was allowed to further chill to ~ 67 °C before samples were added. Next, the RT-LAMP microtubes were incubated in the thermos with the lid tightly closed for 60 min, with photos taken at 30–60 min. During incubation, the microtubes were secured on a foam floater to ensure that they were vertically and sufficiently submerged in water to activate the RT-LAMP reaction. Finally, the tubes were removed from thermos and briefly chilled on ice to allow color stabilization, before being photographed for test result readout.

### Quantitative RT-PCR (OHSU)

Heat-inactivated SARS-CoV-2 RNA was isolated using the Zymo Directzol RNA purification kit according to the manufacturer’s protocol and eluted in 50 μL elution buffer. SARS-CoV2 RNA levels were measured by a one-step quantitative real time reverse transcription polymerase chain reaction assay (qRT-PCR) using TaqMan One-Step RT-PCR Master Mix (Applied Biosystems) with 4 μL per reaction. Primers and probes were as follows: Forward: 5’-TTTGGCTTTGTGTGCTGACTCT; Reverse: 5’-CCCTTTGAGTGCGTGACAAAT and TaqMan probe: 5’ FAM-ATTGGTGGAGCTAAAC-MGB. Forward and reverse primers were used at 250 nM in the reaction, and the probe at 200 nM. For RNA standards, a ten-fold dilution series of 10^6 to 10^1 of a synthetic RNA control (Twist Biosciences: MN908947.3) was used.

### Statistical analysis

To estimate the analytic sensitivity of the RT-LAMP platform with human samples, simulated NP swab samples were spiked with serial dilutions of inactivated WA-1 strain of SARS CoV-2 starting at a calculated 17,473 copies, twofold to 8737 copies and then every 1.6-fold to a final dilution of 227 viral copies. Three independent replicates four-reactions per dilution were performed for each dilution. The average percent-positive for each dilution was calculated and fitted to a dose–response curve with log RNA copy as the dose and percent positive samples as the response and subjected to Find ECanything least squares dose–response curve fitting with lower and upper limits of 0 and 100 respectively (GraphPad Prism V9.3). Fifty and ninety-five percent sensitivity estimates were estimated by setting the F parameter to 50 and 95 respectively.

### Thermoses

Three different types of thermoses were used in these assays, available from Amazon (ASINs B00IR77HMW (#1), B08LPZZGCT (#2), B07MJR3P1H (#3)). Temperature drift experiments were conducted—thermoses were pre-warmed and filled with 67 °C water, lids were secured, and final temperatures taken after 40 min, on 3 separate days. Final temperatures were 61.5 °C, 62.5 °C and 62.5 °C for thermos #1, 61 °C, 65 °C and 65 °C for thermos #2, and 61 °C, 62 °C and 62 °C for thermos #3.

### Instruction for sample self-collection using anterior nasal swab

No food or drink other than water 30 min prior to sample collection. Wash hands prior to sample collection. Insert swab into nostril just enough so the cotton tip is no longer visible. Swipe the inside of nostril in a circular motion, 5 times. Repeat for the other nostril, using the same swab. Dunk the swab into the labeled tube, plunging it into the liquid about 10 times. Discard the swab and replace the lid on the tube.

### Instruction for sample self-collection using gingival swab

No food or drink other than water 30 min prior to sample collection. Wash hands prior to sample collection. Insert bristled swab into mouth and position the swab so that it covers the gingival line (line between gum and teeth) of top teeth. Gently swipe back and forth several times along the gingival line (in a tooth-brushing motion) on the outside face of top teeth. Flip the swab over and repeat for the outside face of bottom teeth. Dunk the gingival swab into the labeled tube, plunging it into the liquid about 10 times. “Squeeze” out the remaining fluid in the swab by pressing it on the side of the tube like a sponge. Discard the swab and replace the lid on the tube.

### Human research ethics

This study has been reviewed and approved by the Oregon Health and Science University (OHSU) Institutional Review Board (IRB#20114). Informed consent was obtained from subjects upon enrollment.

## Supplementary Information

Supplementary Information.

## Data Availability

All data associated with this study are available in the main text or the Supplementary Materials.
