# Supplementary material for: A lyophilized colorimetric RT-LAMP test kit for rapid, low-cost, at-home molecular testing of SARS-CoV-2 and other pathogens
Source: Sci Rep. 2022 Apr 29;12:7043. doi: 10.1038/s41598-022-11144-5 (PMC9052177; doi:10.1038/s41598-022-11144-5)
Supplement: Supplementary file 1 — Supplementary Information. [file 41598_2022_11144_MOESM1_ESM.pdf]

Supplementary Materials for

A Lyophilized Colorimetric RT-LAMP Test Kit for Rapid, Low-Cost, At-Home Molecular Testing of SARS-CoV-2 and Other Pathogens

Xin Song[1,2,3]*, Felicity J. Coulter[4]†, Ming Yang[3]†, Jessica L. Smith[5], Fikadu G. Tafesse[4], William B. Messer[4,6,7]*, John H. Reif[1,3]*

[1] Department of Electrical and Computer Engineering, Duke University, Durham, NC 27708, USA
[2] Department of Biomedical Engineering, Duke University, Durham, NC 27708, USA
[3] Department of Computer Science, Duke University, Durham, NC 27708, USA
[4] Department of Molecular Microbiology & Immunology, Oregon Health & Science University, Portland, OR 97239, USA
[5] Vaccine and Gene Therapy Institute, Oregon Health & Science University, Beaverton, OR 97006, USA
[6] Department of Medicine, Division of Infectious Diseases, Oregon Health & Science University, Portland, OR 97239, USA
[7] Program in Epidemiology, OHSU-PSU School of Public Health, Oregon Health & Science University, Portland, OR 97239, USA

† These authors contributed equally to this work.
* Correspondence to: xin.song@duke.edu, messer@ohsu.edu, reif@cs.duke.edu

Table S1. Detailed cost breakdown of the RT-LAMP home test kit.

Kit component	Item	Vendor	Catalog	Price	Quantity/kit	Cost/kit
Lyophilized RT-LAMP test in single microtube	WarmStart® colorimetric LAMP 2X master mix (DNA & RNA)	New England BioLabs	M1800L	$640.64	10 µL	$1.025
	RT-LAMP primer: F3	IDT	100 nmole DNA Oligo, standard desalting	$6.30	0.004 nmole	$2.52e-4
	RT-LAMP primer: B3	IDT	100 nmole DNA Oligo, standard desalting	$7.70	0.004 nmole	$3.08e-4
	RT-LAMP primer: FIP	IDT	100 nmole DNA Oligo, standard desalting	$17.85	0.032 nmole	$5.712e-3
	RT-LAMP primer: BIP	IDT	100 nmole DNA Oligo, standard desalting	$17.15	0.032 nmole	$5.488e-3
	RT-LAMP primer: LoopF	IDT	100 nmole DNA Oligo, standard desalting	$9.80	0.008 nmole	$7.84e-4
	RT-LAMP primer: LoopB	IDT	100 nmole DNA Oligo, standard desalting	$9.45	0.008 nmole	$7.56e-4
	D-(+)-trehalose dihydrate	Sigma-Aldrich	T9531-25G	$128.00	3.405 mg	$0.017
	Guanidine hydrochloride	VWR	TCG0197-025G	$17.04	0.076 mg	$5.21e-5
	0.2 mL PCR tube	Sarstedt	72.737.002	$19.45	1	$0.039
Sample collection & viral RNA isolation	Sample collection tube	VWR	89005-596	$111.52	1	$0.112
	Anterior nasal swab	Thomas Scientific	MSC-93050D	$35.00	1	$0.35
	Gingival swab	Amazon	B077WQZRTB	$12.99	1	$0.13
	20 µL exact volume transfer pipette	Thomas Scientific	783NL	$62.14	1	$0.124
	Nuclease-free water	Sigma-Aldrich	W4502-1L	$45.76	3 mL	$0.137
	10X Tris-Borate-EDTA (TBE)	Sigma-Aldrich	SRE0062-1L	$72.80	0.75 µL	$5.46e-5
Test kit package & storage	Mylar bag (3x4")	Amazon	B07YM59TDZ	$6.08	1	$0.06
	Silica gel packet	Electron Microscopy Sciences	71206-01	$15.50	1	$0.16
Accessory*	Temperature sticker	Digi-Sense	UX-09035-52	$12.37	1	$1.24
**Total cost per test kit (for anterior nasal sample):**				With accessory:		$3.28
				**Without accessory:**		**$2.04**
**Total cost per test kit (for gingival sample):**				With accessory:		$3.06
				**Without accessory:**		**$1.82**

*Alternatively use a thermometer instead. Other items required for the test but not included in the kit: thermos, ice.

Table S2. SARS-CoV-2 RT-LAMP primer set from the Color Genomics EUA.

Primer	Sequence 5' → 3'
F3	AACACAAGCTTTCGGCAG
B3	GAAATTTGGATCTTTGTCATCC
FIP	TGCGGCCAATGTTTGTAATCAGCCAAGGAAATTTTGGGGAC
BIP	CGCATTGGCATGGAAGTCACTTTGATGGCACCTGTGTAG
LoopF	TTCCTTGTCTGATTAGTTC
LoopB	ACCTTCGGGAACGTGGTT

Table S3. Information of SARS-CoV-2 viruses used in this study.

WHO name	Pango lineage	Location first isolated	Source
-	A	WA, US WA1/2020	BEI, NR-52281
Alpha	B.1.1.7	UK	BEI, NR-54011
Beta	B.1.351	South Africa	BEI, NR-54008
Beta	B.1.351	South Africa	BEI, NR-54009
Delta	B.1.617.2	India	Clinical isolate
Epsilon	B.1.427	CA, US	Clinical isolate
Epsilon	B.1.429	CA, US	Clinical isolate
Iota	B.1.526.2	NY, US	BEI, NR-55359
Kappa	B.1.617.1	India	BEI, NR-55486
Zeta	P.2	Brazil	Clinical isolate
	B.1.2	US	Clinical isolate

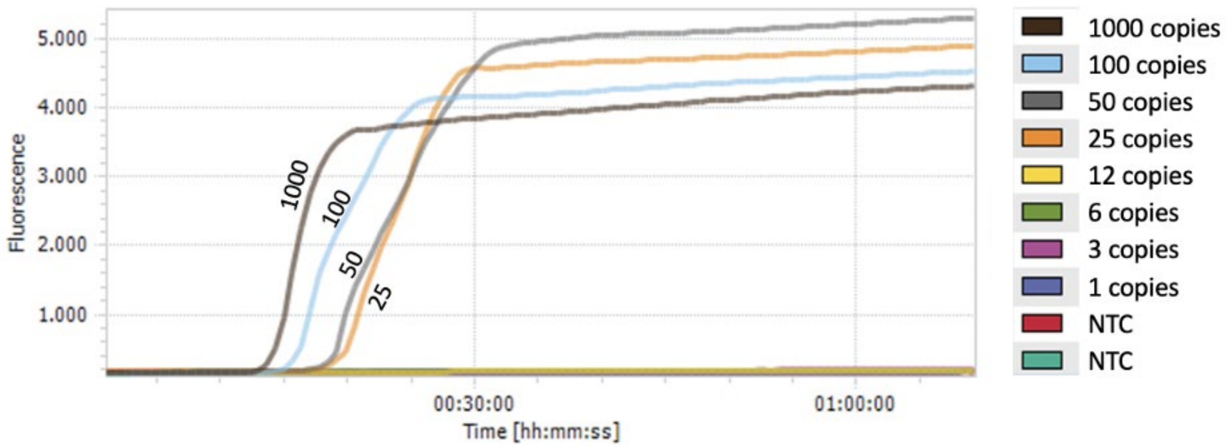

Fig. S1. Initial testing of the NEB primer set[1] by real-time LAMP. Fluorescence curves shown for single dilution series including two none template controls. Reactions done in solution. IDT gBlocks™ gene fragment was used as a proxy target template because SARS-CoV-2 control RNA was not commercially available at the time of this experiment. Copy number indicates total copies of target template per reaction.

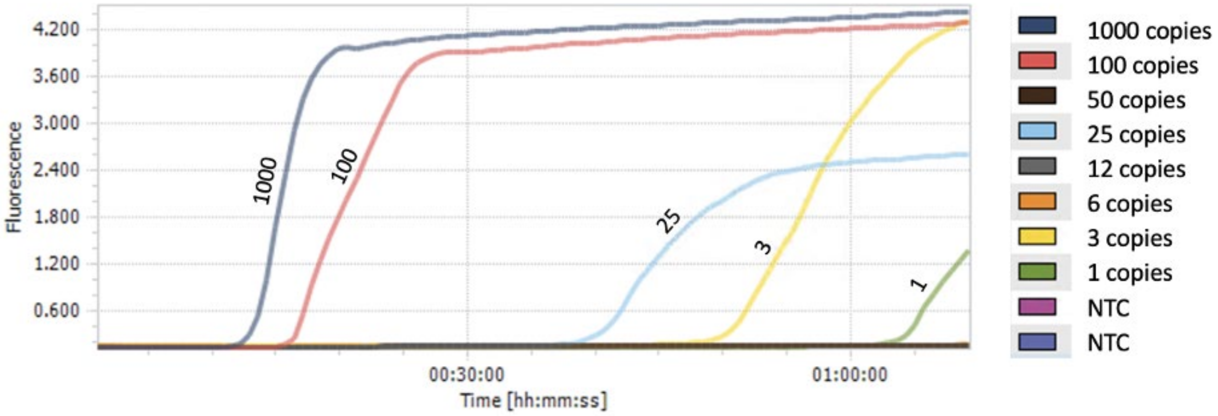

Fig. S2. Initial testing of the Shenyang primer set[2] by real-time LAMP. Fluorescence curves shown for single dilution series including two none template controls. Reactions done in solution. IDT gBlocks™ gene fragment was used as a proxy target template because SARS-CoV-2 control RNA was not commercially available at the time of this experiment. Copy number indicates total copies of target template per reaction.

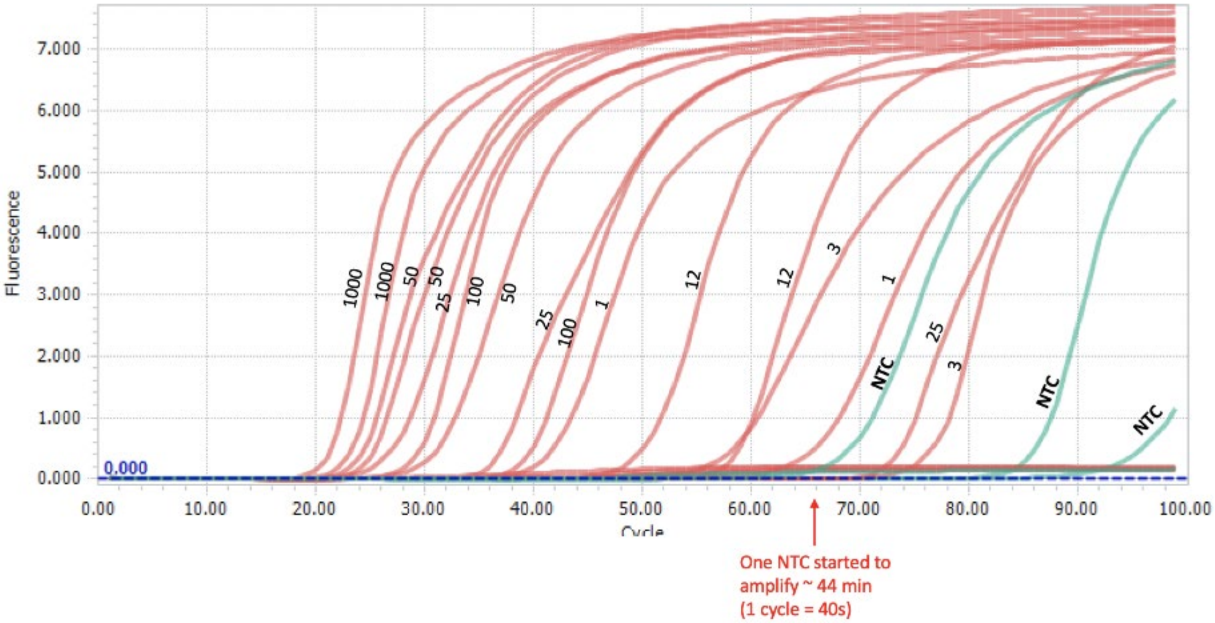

Fig. S3. Further testing of the NEB primer set[1] by real-time LAMP. Amplification curves (with background corrected) shown for duplicate serial dilutions including four none template controls. Reactions done with lyophilized reagents (preliminary formulation before optimization). IDT gBlocks^{TM} gene fragment was used as a proxy target template because SARS-CoV-2 control RNA was not commercially available at the time of this experiment. Copy number indicates total copies of target template per reaction.

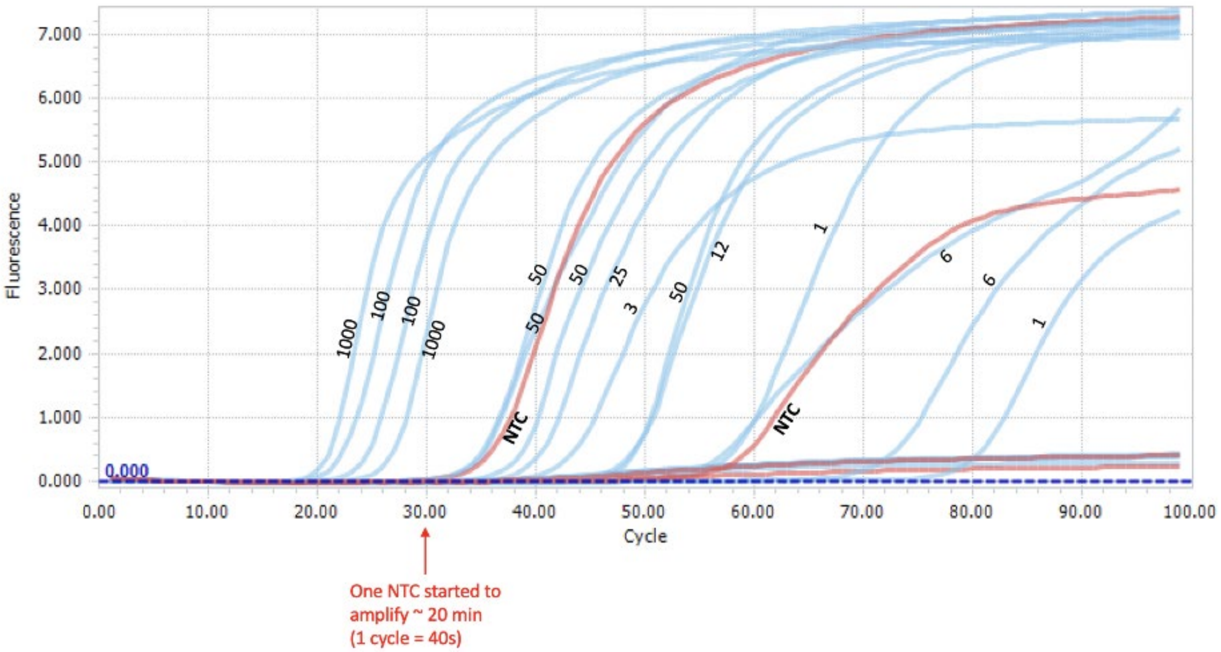

Fig. S4. Further testing of the Shenyang primer set[2] by real-time LAMP. Amplification curves (with background corrected) shown for duplicate serial dilutions including four none template controls. Reactions done with lyophilized reagents (preliminary formulation before optimization). IDT gBlocks^{TM} gene fragment was used as a proxy target template because SARS-CoV-2 control RNA was not commercially available at the time of this experiment. Copy number indicates total copies of target template per reaction.

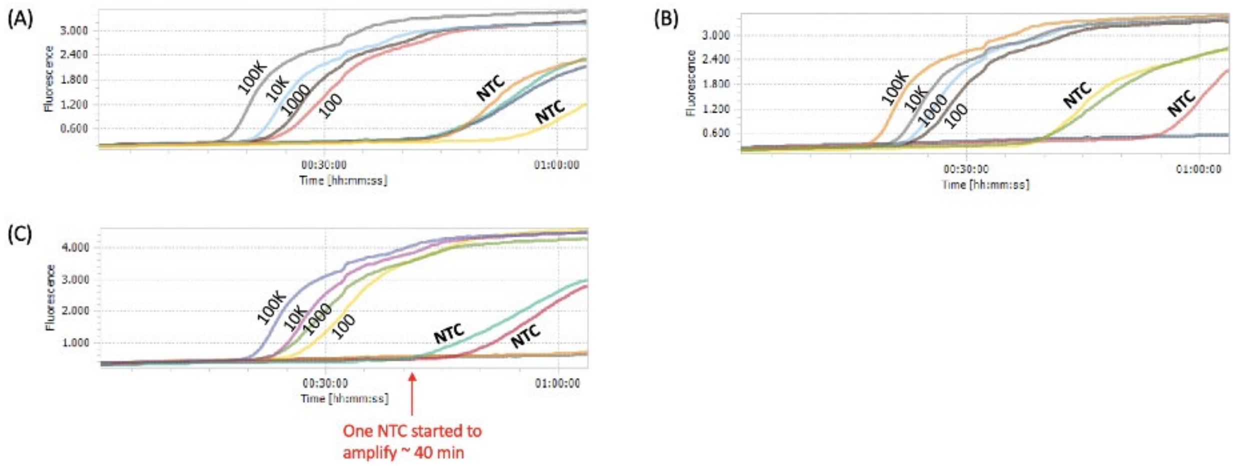

Fig. S5. Initial testing of the New York primer set[3] by RT real-time LAMP. In each panel, fluorescence curves are shown for a single dilution series including two non-template controls. Target template was SARS-CoV-2 synthetic control RNA from Twist Biosciences. Copy number indicates total copies of target template per reaction. (A) Solution-based RT-LAMP, 1st replicate. (B) Solution-based RT-LAMP, 2nd replicate. (C) RT-LAMP from lyophilized reagents (preliminary formulation before optimization).

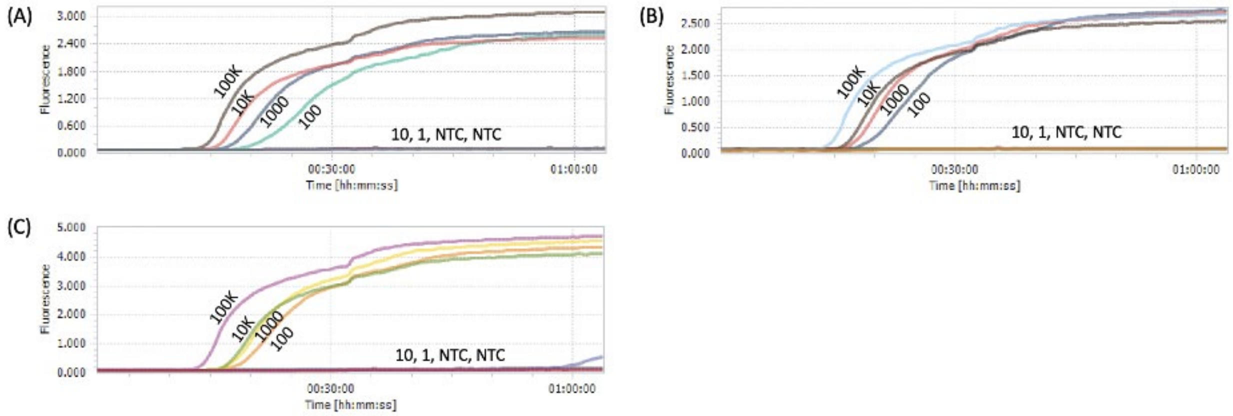

Fig. S6. Initial testing of the Harvard primer set[4] by RT real-time LAMP. In each panel, fluorescence curves are shown for a single dilution series including two non-template controls. Target template was SARS-CoV-2 synthetic control RNA from Twist Biosciences. Copy number indicates total copies of target template per reaction. (A) Solution-based RT-LAMP, 1st replicate. (B) Solution-based RT-LAMP, 2nd replicate. (C) RT-LAMP from lyophilized reagents (preliminary formulation before optimization).

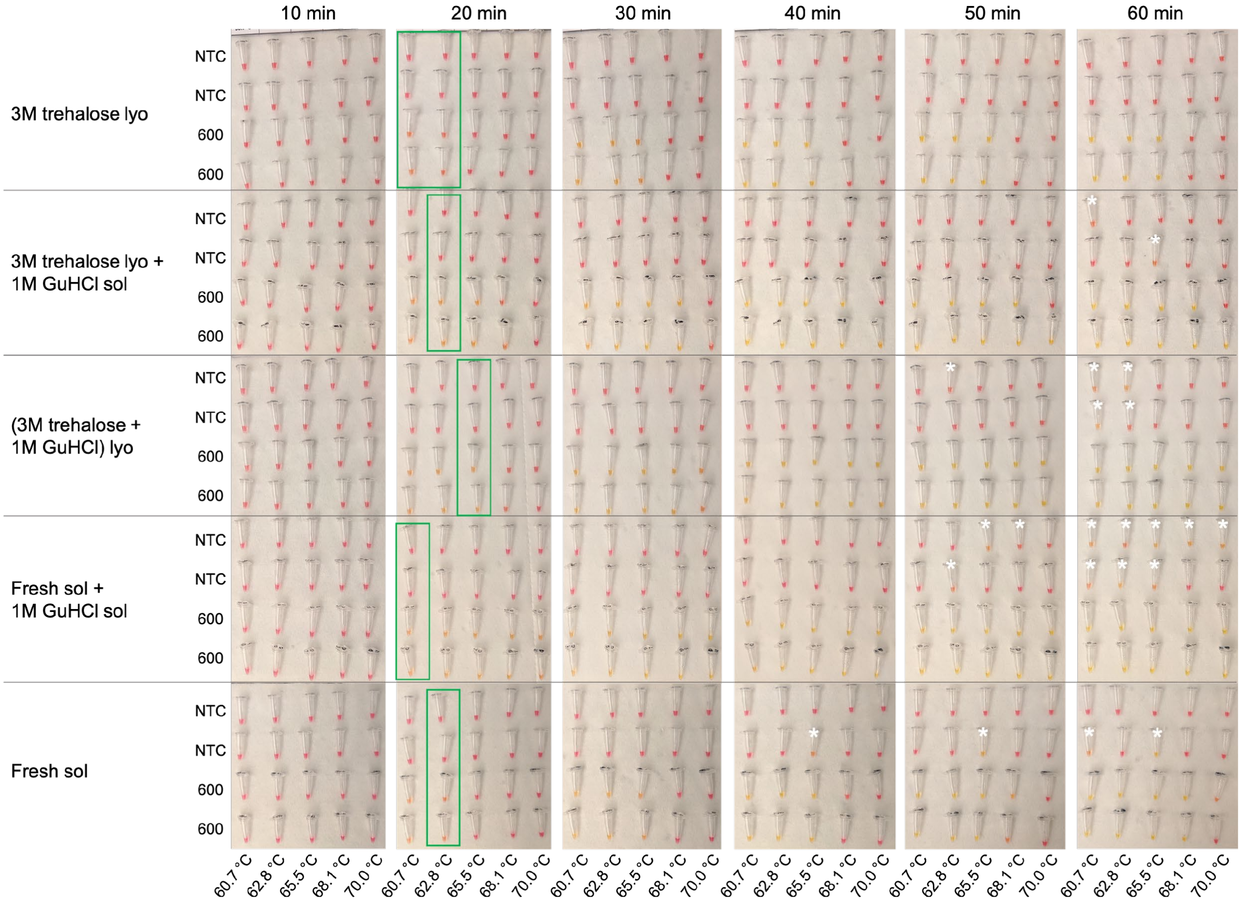

Fig. S7. Comparison and optimization of different RT-LAMP formulations by thermal gradient test. All lyophilized (lyo) samples were stored at room temperature for 1 day before the RT-LAMP experiment. Fresh samples in solution (sol) were prepared at the time of the RT-LAMP experiment. For each set, the optimal incubation temperature was determined as the temperature that achieved both fast reaction (i.e., short time to true positives) and minimal false positives (i.e., long separation between true positives and false positives). NTC refers to none template control. 600 refers to 600 total copies of synthetic SARS-CoV-2 RNA per reaction. False positives are marked by white asterisks. RT-LAMP reactions with optimal temperatures are shown in green boxes.

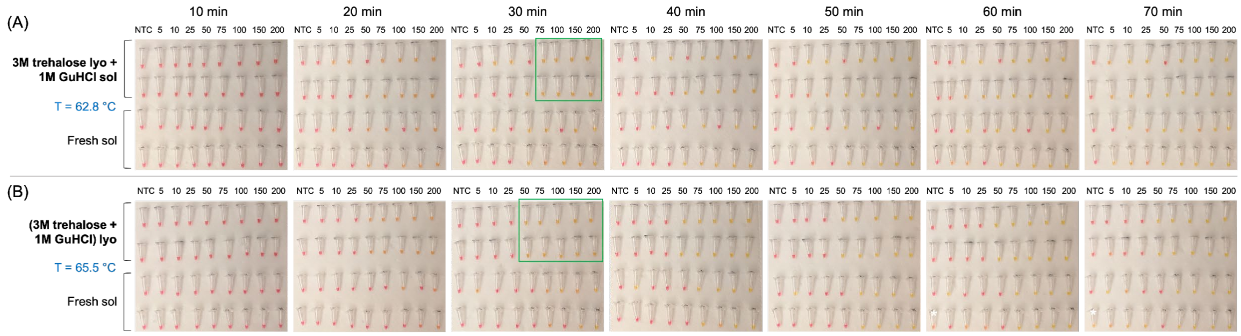

Fig. S8. Analytical sensitivity comparison of two lyophilized RT-LAMP formulations. (A) 3M lyo + GuHCl sol. (B) (3M + GuHCl) lyo. All lyo samples were stored at room temperature for 1 day before the RT-LAMP experiment. Fresh samples were prepared on the day of the RT-LAMP experiment and were included in the experiment as reference. Each reaction was incubated at the indicated optimal temperature. NTC refers to none template control. Column numbers indicate total RNA copies per 20 µL reaction. False positives are marked by white asterisks.

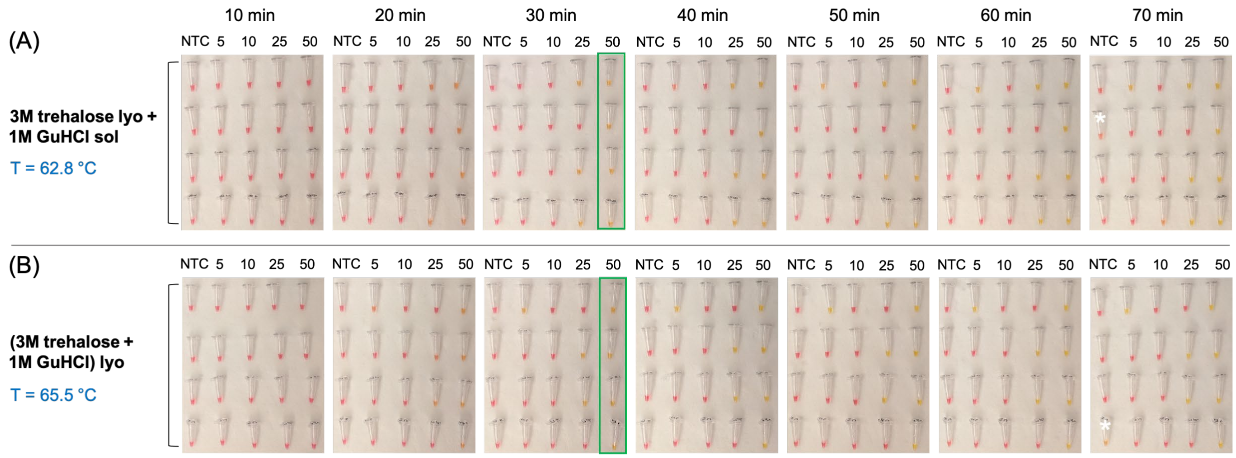

Fig. S9. Further sensitivity analysis of two lyophilized RT-LAMP formulations. (A) 3M lyo + GuHCl sol. (B) (3M + GuHCl) lyo. All lyo samples were stored at room temperature for 1 day before the RT-LAMP experiment. Each reaction was incubated at the indicated optimal temperature. NTC refers to none template control. Column numbers indicate total RNA copies per 20 µL reaction. False positives are marked by white asterisks.

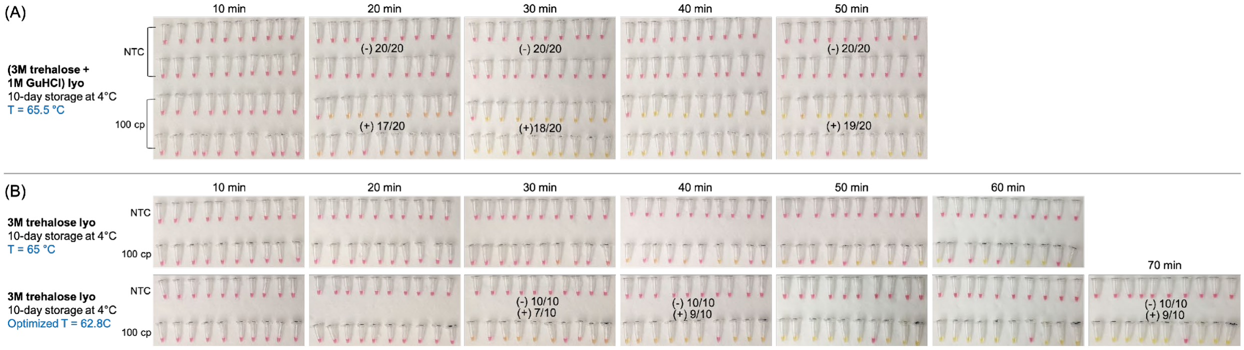

Fig. S10. Lyophilized test kit performance after 10-day storage in fridge (4 °C). (A) Analytical validation of test sensitivity and specificity for the optimal (3M + GuHCl) lyo formulation. (B) Performance of the test lyophilized with 3M trehalose without GuHCl. NTC refers to none template control. 100 cp refers to 100 total copies of RNA per 20 µL reaction. True negatives and true positives are labeled by (-) and (+), respectively. The importance of temperature optimization is apparent as shown by results in (B).

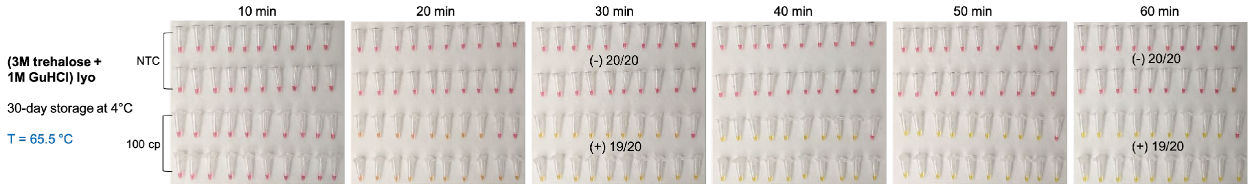

Fig. S11. Lyophilized test kit performance after 30-day storage in the fridge (4 °C). Analytical validation of test sensitivity and specificity for the optimal (3M + GuHCl) lyo formulation. NTC refers to none template control. 100 cp refers to 100 total copies of RNA per 20 µL reaction. True negatives and true positives are labeled by (-) and (+), respectively.

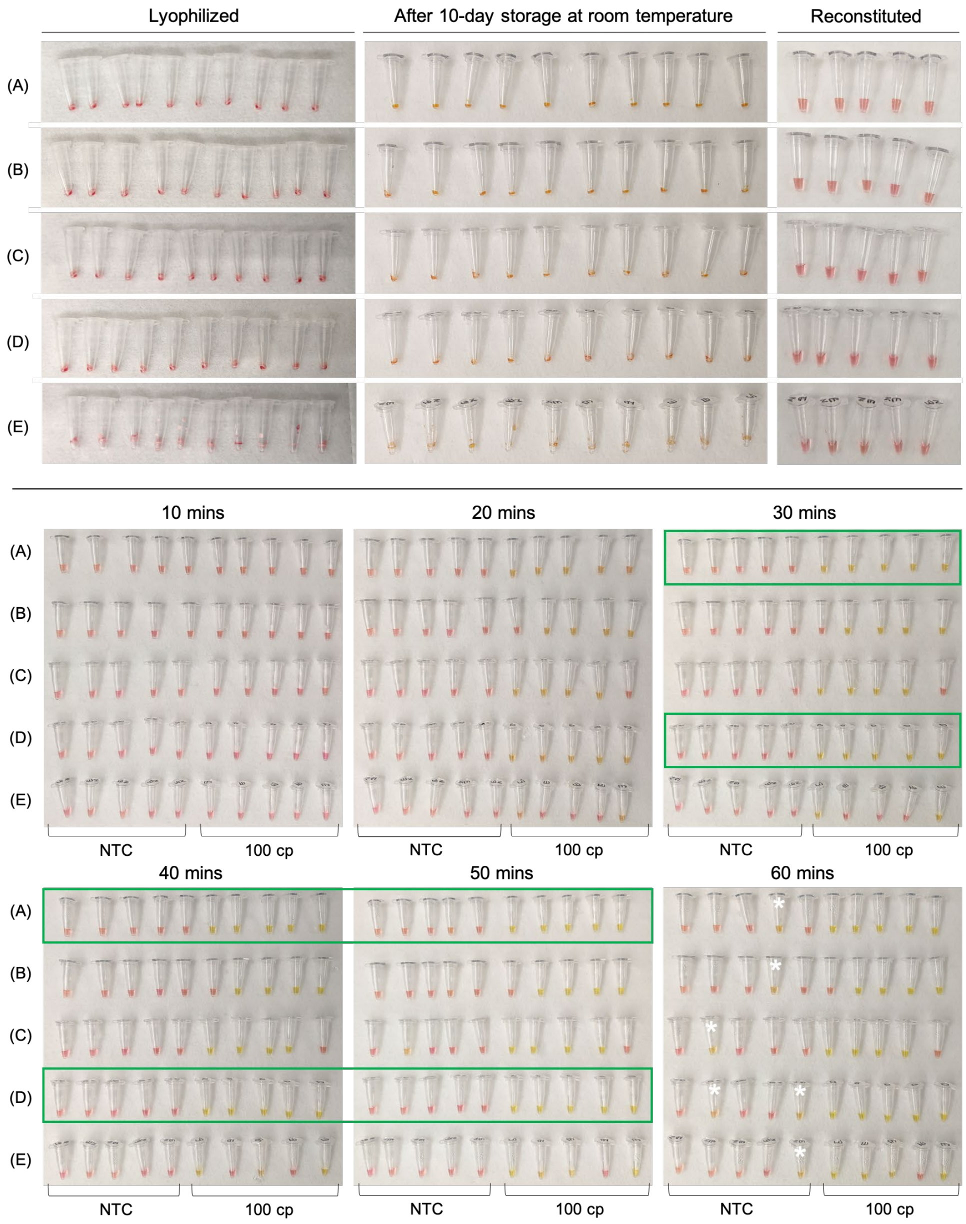

Fig. S12. Lyophilization cycle optimization for extended room temperature storage. (A) 1-hr lyophilization. (B) 1-hr lyophilization with 10-minute 45 °C secondary drying. (C) 1-hr lyophilization with 30-minute 45 °C secondary. (D) 1-hr lyophilization with 1-hr 45 °C secondary drying. (E) 1-hr lyophilization without using vacuum concentrator. (A) to (D) were carried out in a vacuum concentrator connected to the lyophilizer. Top panel shows the appearance of the RT-LAMP test kit after lyophilization, storage, and reconstitution. As there was no color difference among the reconstituted tubes, only 5 tubes are shown for each set. Bottom panel shows RT-LAMP test results after 10-day storage at room temperature. Best performing sets are labeled by green boxes. NTC refers to none template control. 100 cp refers to 100 total copies of RNA per 20 μL reaction. False positives are marked by white asterisks.

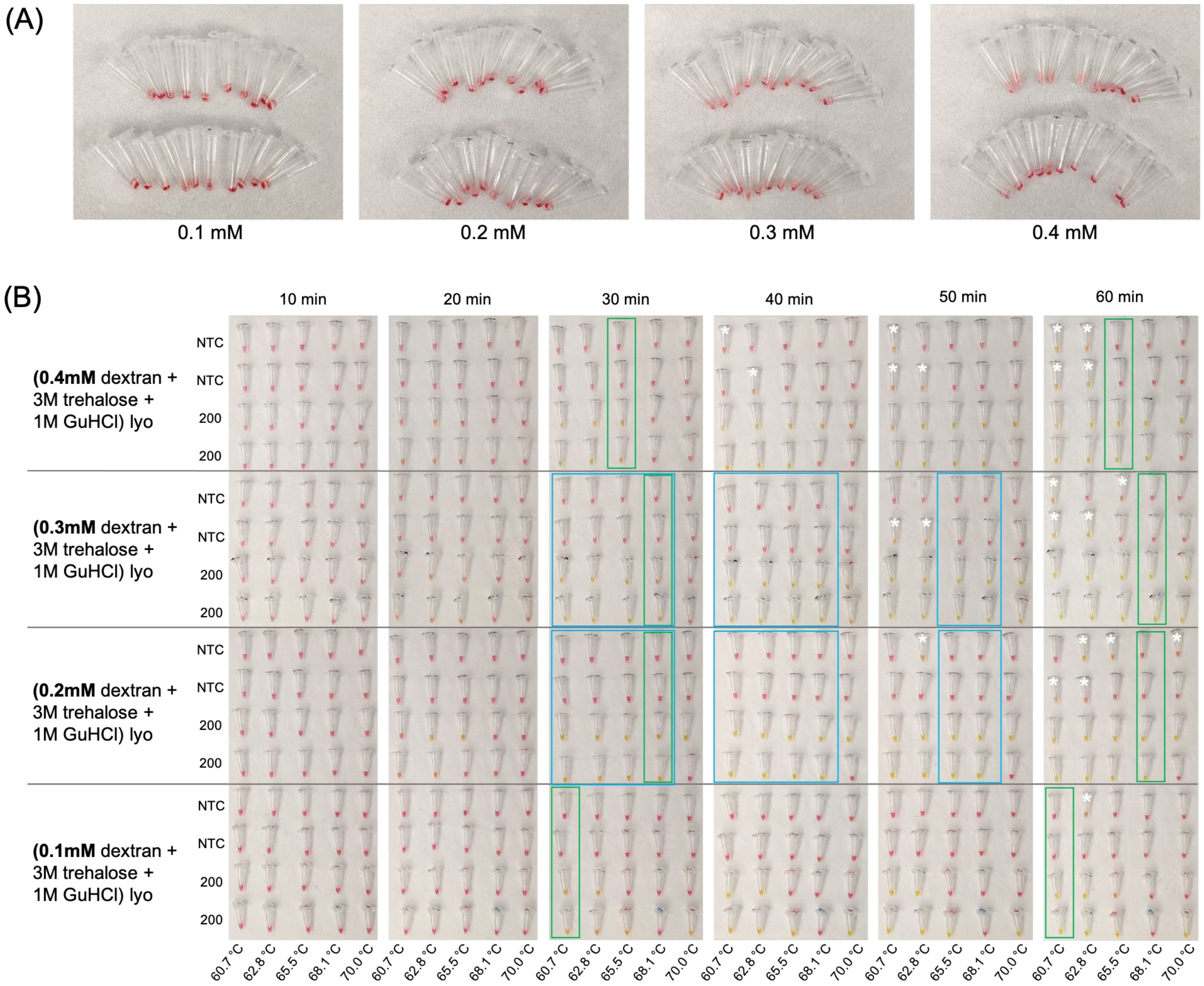

Fig. S13. Lyophilization formulation optimization for extended room temperature storage. (A) Physical appearance of the RT-LAMP reagents lyophilized with dextran added at different concentrations. (B) RT-LAMP tests conducted after storing the lyophilized test kit for 3 days at room temperature. NTC refers to none template control. 200 refers to 200 total copies of RNA per 20 µL reaction. Green boxes indicate RT-LAMP reactions at optimal temperatures with no false positives by 60 minutes. Blue boxes indicate RT-LAMP reactions that tolerate a wide range of temperatures for incubation under 50 minutes. False positives are marked by white asterisks.

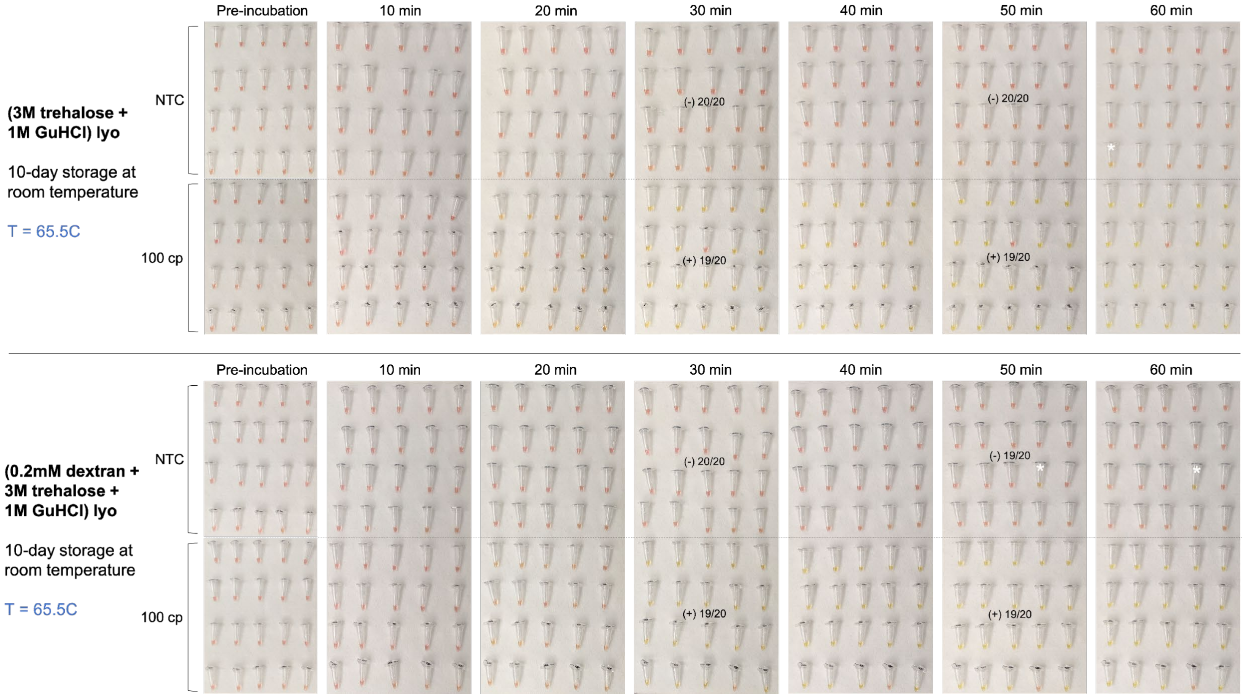

Fig. S14. Comparison of two lyophilization formulations (with and without dextran) for extended room-temperature storage. Lyophilized test kits were stored at room temperature for 10 days and then reconstituted to run RT-LAMP. NTC refers to none template control. 100 cp refers to 100 total copies of RNA per 20 µL reaction. True negatives and true positives are labeled by (-) and (+), respectively. False positives are marked by white asterisks.

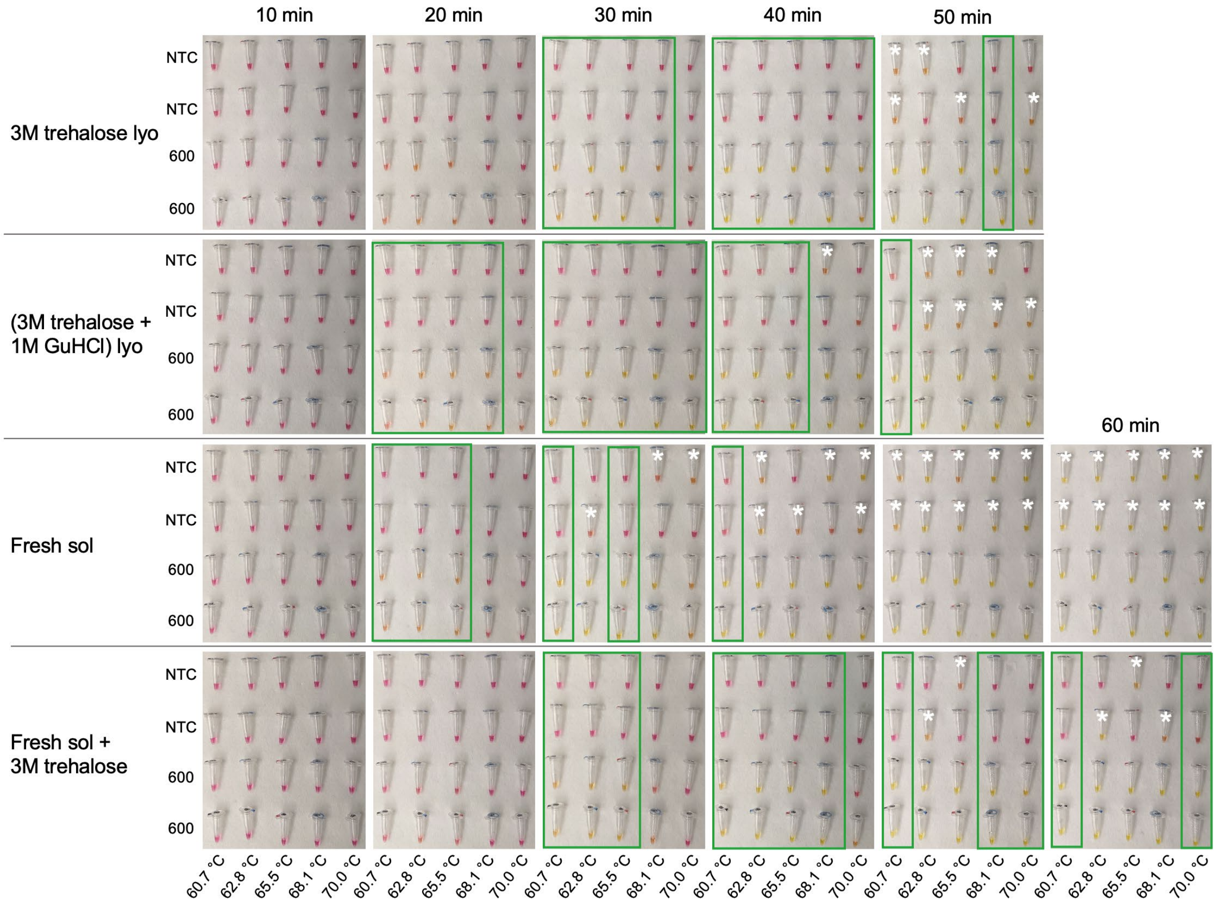

Fig. S15. Comparison and optimization of different RT-LAMP formulations based on Color Genomics primer set. All lyo samples were stored at room temperature for 1 day before the RT-LAMP experiment. Fresh samples were prepared at the time of the RT-LAMP experiment. NTC refers to none template control. 600 refers to 600 total copies of RNA per 20 μL reaction. Green boxes indicate tolerable incubation temperatures with reliable results. False positives are marked by white asterisks. The addition of 3M trehalose drastically reduced the false positives but slightly delayed the time to true positives. The lyophilized RT-LAMP reactions enabled a wider compatible range of incubation temperatures. Overall, the (3M trehalose+1M GuHCl) lyo set was optimal in terms of short time to true positives, wide range of tolerable temperatures, and low false positive rate.

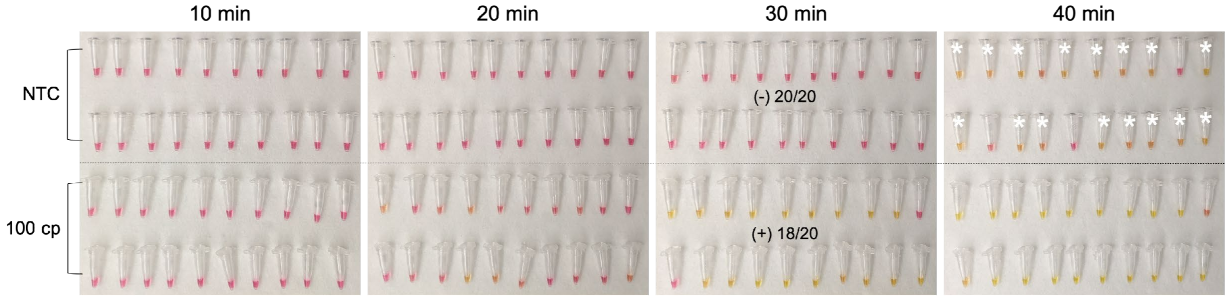

Fig. S16. Performance of the lyophilized test using the Color Genomics primer set after 10-day storage in the fridge (4 °C). RT-LAMP incubation temperature at 65.5 °C. NTC refers to none template control. 100 cp refers to 100 total copies of RNA per 20 μL reaction. True negatives and true positives are labeled by (-) and (+), respectively. False positives are marked by white asterisks.

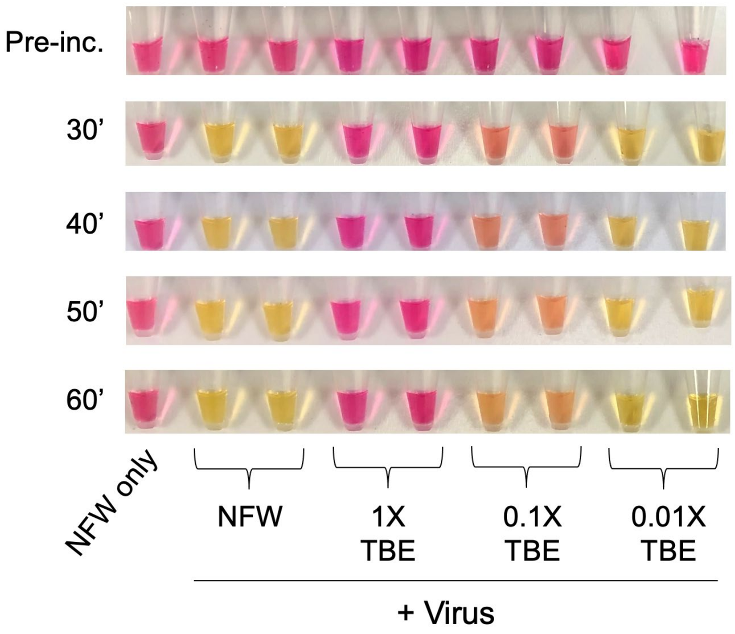

Fig. S17. Optimization of the TBE concentration. Heat-inactivated SARS-CoV-2 virus was diluted in nuclease-free water (NFW), 1X TBE, 0.1X TBE, and 0.01X TBE. Virus-spiked samples were heated at 95 °C for 5 minutes and chilled on ice. Sample input volume was 5 µL for each 20 µL RT-LAMP reaction, to give working concentrations of 0.25X, 0.025X and 0.0025X TBE. According to results shown, a final concentration of 0.0025X TBE achieved the highest sensitivity with colorimetric RT-LAMP.

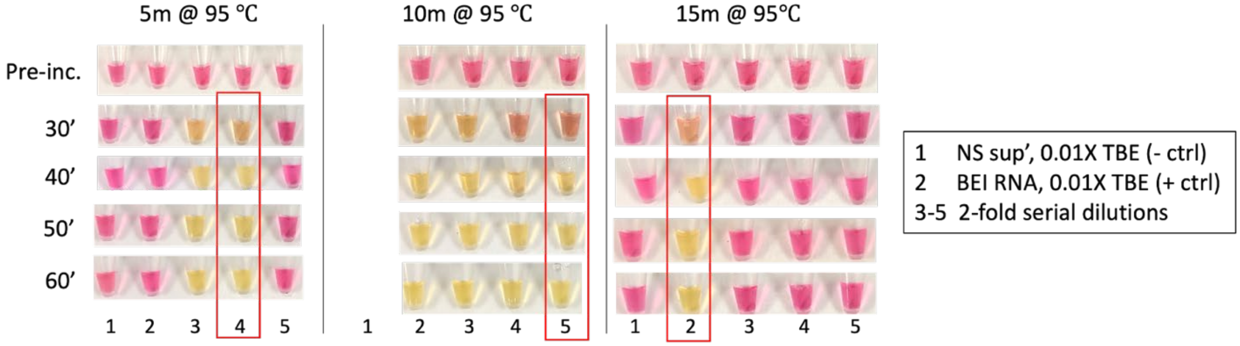

Fig. S18. Optimization of the viral RNA isolation protocol for anterior nasal swab samples. Heat-inactivated SARS-CoV-2 virus was serially diluted (2-fold) to end-point, in 0.01X TBE (Tubes #3-5, decreasing concentration). Virus-spiked samples were heated at 95 °C for 5, 10, or 15 minutes and then chilled on ice. Sample input volume was 5 µL for each 20 µL RT-LAMP reaction. Tube #1 of the 10-min experiment was cracked and discarded, and the positive control (Tube #2) likely failed due to the degradation of RNA after multiple thaws. The 5-, 10- and 15-minute viral isolation assays were set up at the same time, and thus, the other negative controls (5- and 15-minutes) and the positive results from heat-inactivated virus (Tubes #3–5) were deemed sufficient. Highest sensitivity as visualized by colorimetric change in final reactions was achieved by heating at 95 °C for 10 minutes.

Supplementary References

1 Zhang, Y. *et al.* Rapid Molecular Detection of SARS-CoV-2 (COVID-19) Virus RNA Using Colorimetric LAMP. *medRxiv*, 2020.2002.2026.20028373, doi:10.1101/2020.02.26.20028373 (2020).

2 Yu, L. *et al.* Rapid Detection of COVID-19 Coronavirus Using a Reverse Transcriptional Loop-Mediated Isothermal Amplification (RT-LAMP) Diagnostic Platform. *Clinical Chemistry* **66**, 975-977, doi:10.1093/clinchem/hvaa102 (2020).

3 Butler, D. *et al.* Shotgun transcriptome, spatial omics, and isothermal profiling of SARS-CoV-2 infection reveals unique host responses, viral diversification, and drug interactions. *Nature Communications* **12**, doi:10.1038/s41467-021-21361-7 (2021).

4 Rabe, B. A. & Cepko, C. SARS-CoV-2 detection using isothermal amplification and a rapid, inexpensive protocol for sample inactivation and purification. *Proceedings of the National Academy of Sciences of the United States of America* **117**, 24450-24458, doi:10.1073/pnas.2011221117 (2020).
